# Comprehensive analysis of key genes and pathways for biological and clinical implications in thyroid-associated ophthalmopathy

**DOI:** 10.1186/s12864-022-08854-5

**Published:** 2022-09-02

**Authors:** Yueyue Wang, Yanfei Shao, Haitao Zhang, Jun Wang, Peng Zhang, Weizhong Zhang, Huanhuan Chen

**Affiliations:** 1 Department of Endocrinology, The First Affiliated Hospital of Nanjing Medical University, Nanjing, China; 2 Department of General Surgery, Ruijin Hospital, Shanghai Jiao Tong University School of Medicine, Shanghai, China; 3 Shanghai Minimally Invasive Surgery Center, Ruijin Hospital, Shanghai Jiao Tong University School of Medicine, Shanghai, China; 4 Department of Toxicology, The Key Laboratory of Modern Toxicology of Ministry of Education, School of Public Health, Nanjing Medical University, Nanjing, China; 5 Department of Ophthalmology, The Friendship Hospital of Ili Kazakh Autonomous Prefecture Ili & Jiangsu Joint Institute of Health, Ili, China; 6 Department of Ophthalmology, The First Affiliated Hospital of Nanjing Medical University, Nanjing, China

**Keywords:** Thyroid-associated ophthalmopathy, Bioinformatics analysis, Hub gene, Biomarker, Inflammation, Neuromodulation

## Abstract

**Background:**

Thyroid-associated ophthalmopathy (TAO) is a common and organ-specific autoimmune disease. Early diagnosis and novel treatments are essential to improve the prognosis of TAO patients. Therefore, the current work was performed to identify the key genes and pathways for the biological and clinical implications of TAO through comprehensive bioinformatics analysis and a series of clinical validations.

**Methods:**

GSE105149 and GSE185952 were obtained from the Gene Expression Omnibus (GEO) database for analysis. The data were normalized to identify the common differentially expressed genes (DEGs) between the two datasets, and the Gene Ontology (GO) and Kyoto Encyclopedia of Genes and Genomes (KEGG) enrichment analyses were conducted to assess key pathways in TAO. Protein–protein interaction (PPI) networks and hub genes among the common DEGs were identified. Furthermore, we collected the general information and blood samples from 50 TAO patients and 20 healthy controls (HCs), and the expression levels of the proteins encoded by hub genes in serum were detected by enzyme-linked immunosorbent assay (ELISA). Then we further assessed the relationship between the ELISA data and the TAO development.

**Results:**

Several common pathways, including neuroactive ligand-receptor interaction, the IL-17 signaling pathway, and the TNF signaling pathway, were identified in both datasets. In parallel, 52 common DEGs were identified. The KEGG analysis showed that these common DEGs are mainly enriched in long-term depression, the VEGF signaling pathway, the IL-17 signaling pathway, the TNF signaling pathway, and cytokine-cytokine receptor interactions. The key hub genes PRKCG, OSM, DPP4, LRRTM1, CXCL6, and CSF3R were screened out through the PPI network. As confirmation, the ELISA results indicated that protein expression levels of PRKCG, OSM, CSF3R, and DPP4 were significantly upregulated in TAO patients compared with HCs. In addition, PRKCG and DPP4 were verified to show value in diagnosing TAO, and CSF3R was found to be a valuable diagnostic marker in distinguishing active TAO from inactive TAO.

**Conclusions:**

Inflammation- and neuromodulation-related pathways might be closely associated with TAO. Based on the clinical verification, OSM, CSF3R, CXCL6, DPP4, and PRKCG may serve as inflammation- or neuromodulation-related biomarkers for TAO, providing novel insights for the diagnosis and treatment of TAO.

**Supplementary Information:**

The online version contains supplementary material available at 10.1186/s12864-022-08854-5.

## Introduction

Thyroid-associated ophthalmopathy (TAO) is an organ-specific autoimmune disease of the orbit that results in eyelid contracture, exophthalmos, diplopia, corneal ulcerations, and even loss of vision due to optic nerve compression [1]. Previous studies have shown that TAO leads to a significant deterioration in the quality of life [2] and even precipitates the emergence of psychosocial disorders such as depression and anxiety [3, 4]. As the first-line treatment, intravenous glucocorticoids represent the positive therapy for moderate-to-severe and active TAO; however, a completely satisfactory response is rare [5]. In addition, if vision deteriorates progressively, orbital decompression should be performed immediately [6]. However, for individuals who do not respond to orbital decompression, treatment options are very limited. Therefore, early diagnosis and effective therapeutic strategies are essential to improve the prognosis of TAO patients.

With the rapid development of high-throughput sequencing technology and the popularization of public databases, multiple gene expression profiling studies using bioinformatics analysis have been performed to identify new diagnostic or therapeutic biomarkers for different diseases, such as cancers and metabolic diseases and cardiovascular diseases [7–9]. Bioinformatics technology in particular has become an indispensable aspect of identifying key genes and pathways in and exploring the pathogenesis of certain diseases, including TAO. Recently, a few microarray gene profile studies on TAO have been performed. For example, Zhao et al. showed based on gene expression profiling that some genes related to cell cycle, proteasome encoding, and pathways of ribosomes, as well as retinol metabolism, might play a pivotal role in the development of TAO [10]. Through microarray analysis, Chen et al. indicated that the leptin receptor is a key gene positively related to cell adhesion process in TAO immunopathogenesis [11]. Another group addressed the molecular mechanisms of lacrimal gland enlargement in TAO patients and screened some key genes associated with endoplasmic reticulum protein processing of the lacrimal gland through mining the microarray datasets, such as HSP90AA1, HSP90B1, DNAJC10, HSPA5, and CANX [12]. In addition, a study using bioinformatics combined with proteomic and miRNA analysis found some meaningful circulating biomarkers that predict TAO disease status, including zonulin, alpha-2 macroglobulin, beta-2 glycoprotein 1, and fibronectin [13]. All these findings have revealed biomarkers that may be useful to explore the molecular mechanism of TAO. Nevertheless, there are some limitations among these studies, such as examination of a single tissue, lack of independent verification, and incomplete mechanisms of TAO pathogenesis.

In this study, we profiled key genes and potential molecular pathways in different lesion tissues of TAO by bioinformatics analysis using two datasets. To strengthen the accuracy of our results, we collected serum samples from 50 TAO patients and 20 healthy individuals for clinical and molecular biology validation. The novel genes and pathways obtained provide new insight into the underlying mechanisms and reveal a new strategy for the diagnosis and treatment of TAO.

## Materials and methods

### Microarray data

The Gene Expression Omnibus (GEO) database covers the gene expression and corresponding clinical data submitted by research institutions from around the world. Two gene expression profiles (GSE105149 and GSE185952) were downloaded from the GEO database (http://www.ncbi.nlm.nih.gov/geo), containing 23520 and 36146 genes, respectively. The GSE105149 dataset contains gene expression levels of 11 lacrimal gland biopsy/surgical waste tissue samples from TAO patients (*n* = 4) and healthy individuals (*n* = 7) based on the GPL570 (Affymetrix Human Genome U133 Plus 2.0 Array) platform. The GSE185952 dataset includes data on orbital adipose/connective tissue samples from TAO patients who underwent orbital decompression (*n* = 3), with control tissue obtained from the healthy people after plastic surgery (*n* = 3), using the GPL30862 (Agilent-086360 Shbio Human (4*180 K) array) platform. The probes were transformed into the corresponding gene symbol according to the annotation information for the platform.

### Identification of differentially expressed genes

Differentially expressed genes (DEGs) between the two datasets TAO and control samples were screened using the limma (linear models for microarray data) package of R software (version 4.1.0, AT&T Bell Laboratories, USA) [14]. For a gene symbol corresponding to multiple probe IDs, the mean expression value was selected as the expression level of the gene. The data were standardized and filtered to select significant DEGs. A *P* value was less than 0.05 (*P* < 0.05) and an absolute value of log fold change (FC) greater than 0.58 (|log2FC|> 0.58) were considered to indicate differential expression. Next, the up-regulated and down-regulated DEGs in the two datasets were imported into Venny 2.1.0 (https://bioinfogp.cnb.csic.es/tools/venny/), and the intersection of the up-regulated and down-regulated DEGs in the two datasets was taken.

### Pathway enrichment analysis of differentially expressed genes

Gene Ontology (GO) biological process terms [15] and Kyoto Encyclopedia of Genes and Genomes (KEGG) pathway enrichment analyses [16] were performed for the DEGs by using the Database for Annotation, Visualization, and Integration Discovery (DAVID, https://david.ncifcrf.gov/) [17] and OmicShare [18] (https://www.omicshare.com/tools) online tools to gain insight into the biological meaning of the common DEGs. GO analysis consists of biological process (BP), cellular component (CC), and molecular function (MF) categories. A value of *P* < 0.05 was considered statistically significant.

### Construction of a protein–protein interaction network and hub gene identification

A PPI network of DEGs was established using Search Tool for the Retrieval of Interacting Genes (STRING, http://www.string-db.org/) [19] and visualized by Cytoscape software (version 3.9.0). The cytoHubba plug-in [20] algorithms maximum neighborhood component (MNC), maximal clique centrality (MCC), and degree values were used to further screen hub genes from the PPI network.

### Clinical sample collection

Fifty patients diagnosed with TAO based on the Bartley criteria [21] between December 2019 and December 2021 at the First Affiliated Hospital of Nanjing Medical University were included in this study. Patients were excluded if they had other autoimmune inflammatory disorders, had been treated with steroids or immunosuppressants, or had undergone radiotherapy, surgical decompression, or thyroidectomy. Patients with a clinical activity score greater than or equal to 3 were defined as active TAO [22]. The patients' medical history and laboratory parameters were collected. The control group consisted of twenty healthy volunteers matched for age and sex with the study group, with no history of thyroid disease, autoimmune disorders, orbital diseases, or immunosuppressive therapy. Two milliliters of cubital venous blood were drawn in the morning when the subjects were fasting. After centrifugation (3000 rpm for 7 min), the serum was stored at -80 ℃. The study protocol was approved by the Ethics Committee of the First Affiliated Hospital of Nanjing Medical University, and all participants signed an informed consent form.

### Enzyme-linked Immunosorbent Assay (ELISA)

ELISA is a commonly used biochemistry assay, first presented by Peter Perlmann and Eva Engvall in 1971 [23]. A solid-phase type of enzyme immunoassay (EIA) is used to detect the presence of a ligand (commonly a protein) in a liquid sample. Its principle of action is using antigen–antibody interactions against the protein to be measured. Expression levels of Protein Kinase C Gamma (PRKCG), Oncostatin M (OSM), Dipeptidyl Peptidase 4 (DPP4), C-X-C Motif Chemokine Ligand 6 (CXCL6), and Colony Stimulating Factor 3 Receptor (CSF3R) in serum were analyzed with ELISA kits (Human PRKCG/Protein kinase C gamma type ELISA Kit, E8823 h, EIAab, China; Human OSM/Oncostatin M ELISA Kit, EK0478, BOSTER, China; Human CD26/DPP4 ELISA Kit, EK0696 BOSTER, China; Human CXCL6/C-X-C motif chemokine 6 ELISA Kit, E1570 h, EIAab, China; Human CSF3R/G-CSF R ELISA Kit, EK0766, BOSTER, China). First, all serum samples were diluted and incubated in microplates. Then the samples were incubated with biotin-labelled antibody after washing three times and subsequently incubated with avidin-labelled horseradish peroxidase and finally with substrate solution. Absorbance values at 450 nm for all samples were assessed using a microplate reader (DENLEY DRAGON Wellscan MK 3, Thermo, Finland). Finally, concentrations of PRKCG, OSM, DPP4, CXCL6, and CSF3R were calculated based on the standard curves of each ELISA plate.

### Statistical analysis

The SPSS software (version 23.0; IBM) and R software were used for statistical analyses. Numerical data are expressed as means ± standard deviation. Differences in quantitative data between the two groups were compared using nonparametric tests or t-tests based on the data distribution characteristics. Categorical variables were compared using the chi-square test. Spearman's rank correlation coefficient was used to evaluate the correlation between expression protein expression level and clinical characteristics. Multivariate logistic regression analysis was performed to explore independent associated factors of TAO. The diagnostic value of hub gene expression levels in TAO was determined by receiver operating characteristic (ROC) curve analysis and area under the curve (AUC) calculation. *P* values < 0.05 were considered statistically significant.

## Results

### Identification of DEGs

**Fig. 1:**
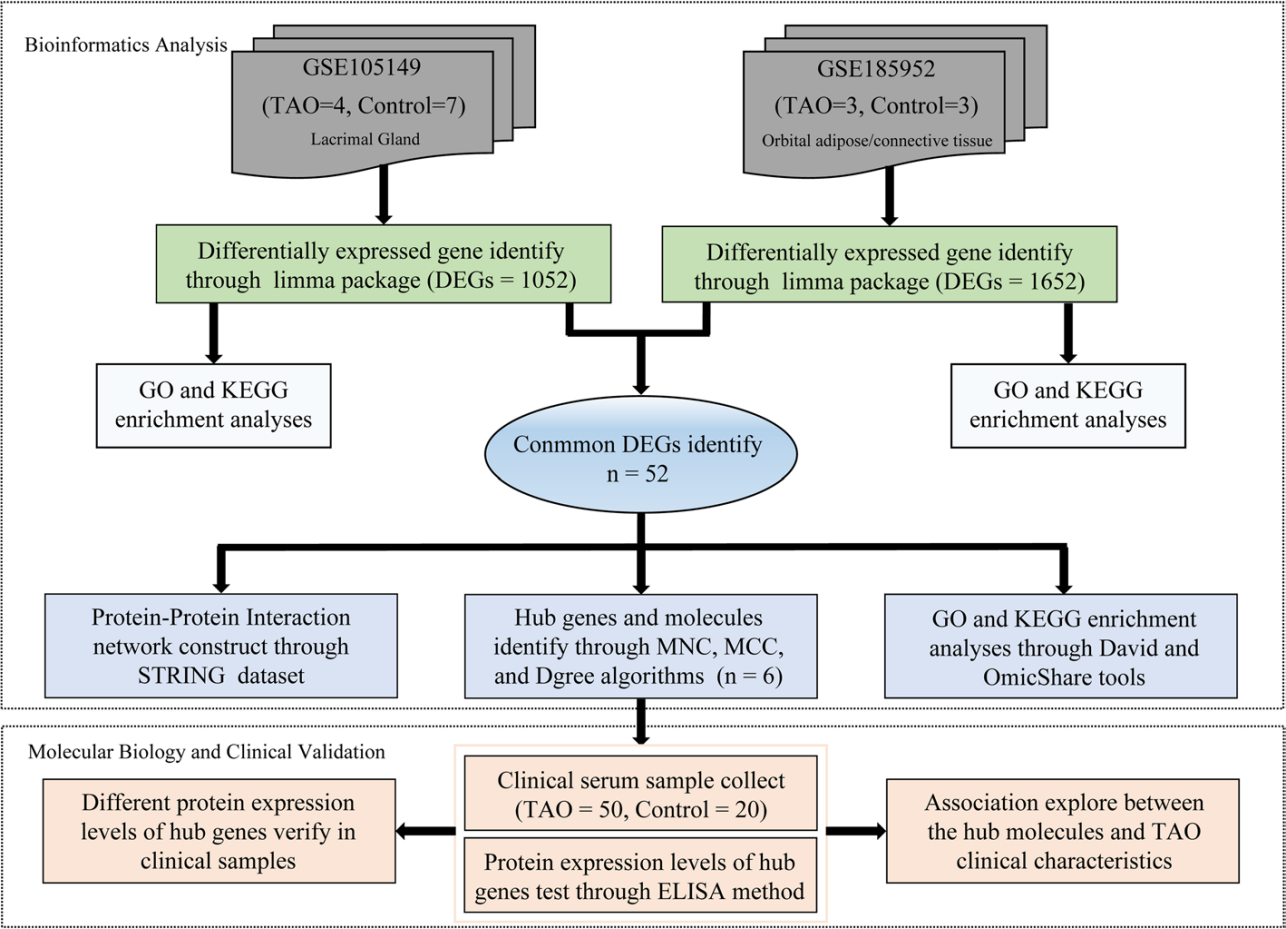
Complete procedures of our research

**Fig. 2:**
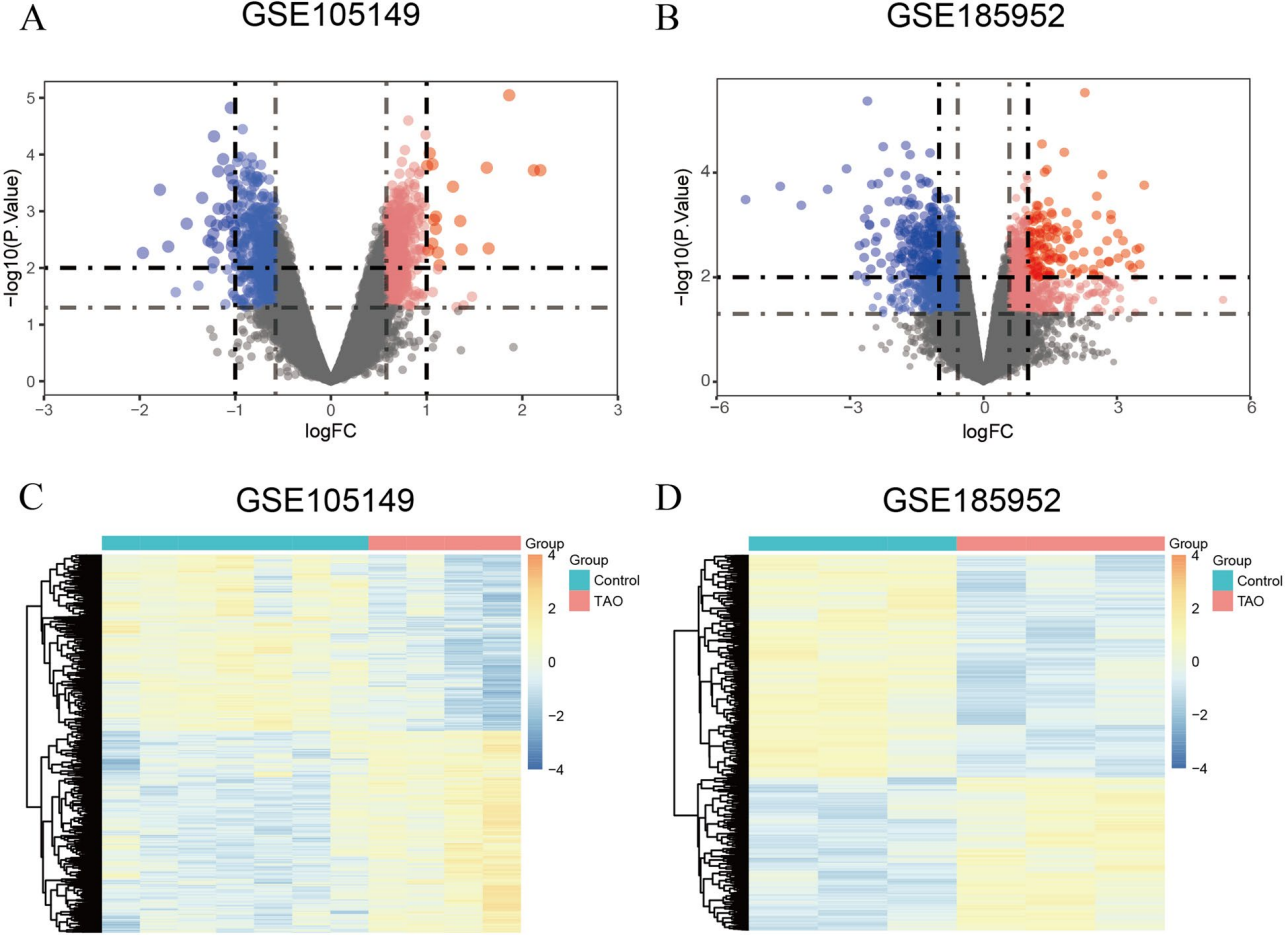
Identification of the DEGs. **A** and **B** The volcano plot of all DEGs in GSE105149 and GSE185952 datasets. Red and blue nodes represent up-regulated genes and down-regulated genes, respectively. **C** and **D** The heat maps showed the expression profiles of the DEGs in GSE105149 and GSE185952 datasets

The overall design of the study is shown in Fig. 1. After checking and screening genes without matched gene symbols, 554 genes were up-regulated and 498 genes were down-regulated in the GSE105149 dataset. We found that 659 genes were up-regulated and 993 genes down-regulated in the GSE185952 dataset. The differences in the gene expression levels in the GSE105149 (Fig. 2A, C) and GSE185952 (Fig. 2B, D) datasets are displayed as volcano and heatmap plots.

### Analysis of functional enrichment

**Fig. 3:**
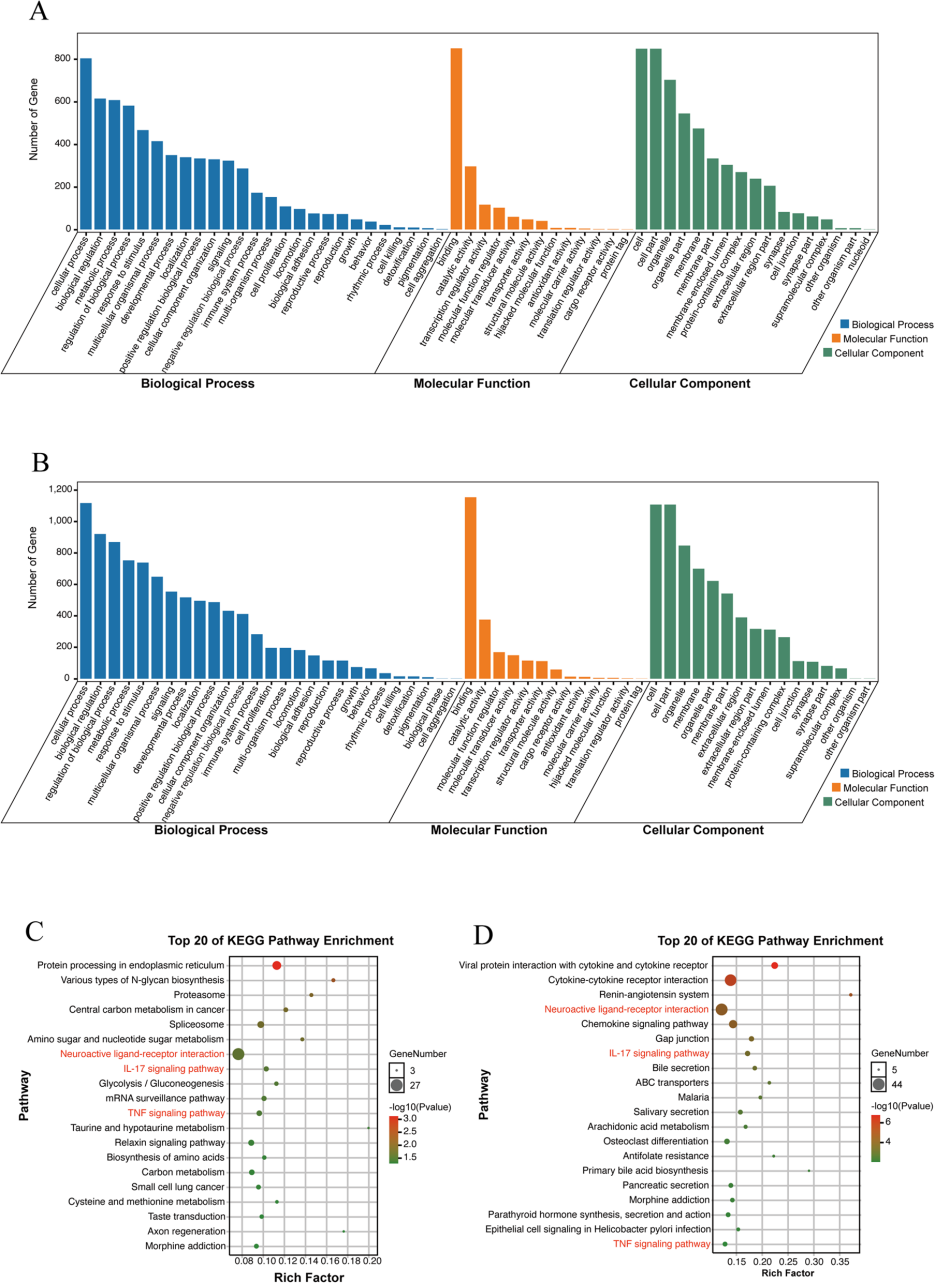
GO and KEGG enrichment analysis of the DEGs. **A** and **B** GO annotation of the DEGs in GSE105149 and GSE185952. **C** and **D** KEGG enrichment results of the DEGs in GSE105149 and GSE185952

To explore molecular biological functions and pathways in TAO, the GO enrichment and KEGG pathway analysis were performed on DEGs between the TAO and healthy samples in the GSE105149 and GSE185952 datasets. BP, MF and CC were all covered in the GO analysis, and the results for the GSE105149 (Fig. 3A) and GSE185952 (Fig. 3B) datasets are shown as bar plots. The GO enrichment results indicated the DEGs to be enriched in several common molecular functions, such as biological regulation and metabolic process for BP, catalytic activity, and transcription regulator activity for MF, and organelle and membrane for CC in both datasets. In KEGG pathway analysis, several common pathways were found to be enriched in both datasets, including neuroactive ligand-receptor interaction, IL-17 signaling pathway, and tumor necrosis factor (TNF) signaling pathway (Fig. 3C, D).

### Identification and analysis of the common DEGs between the GSE105149 and GSE185952 datasets

**Fig. 4:**
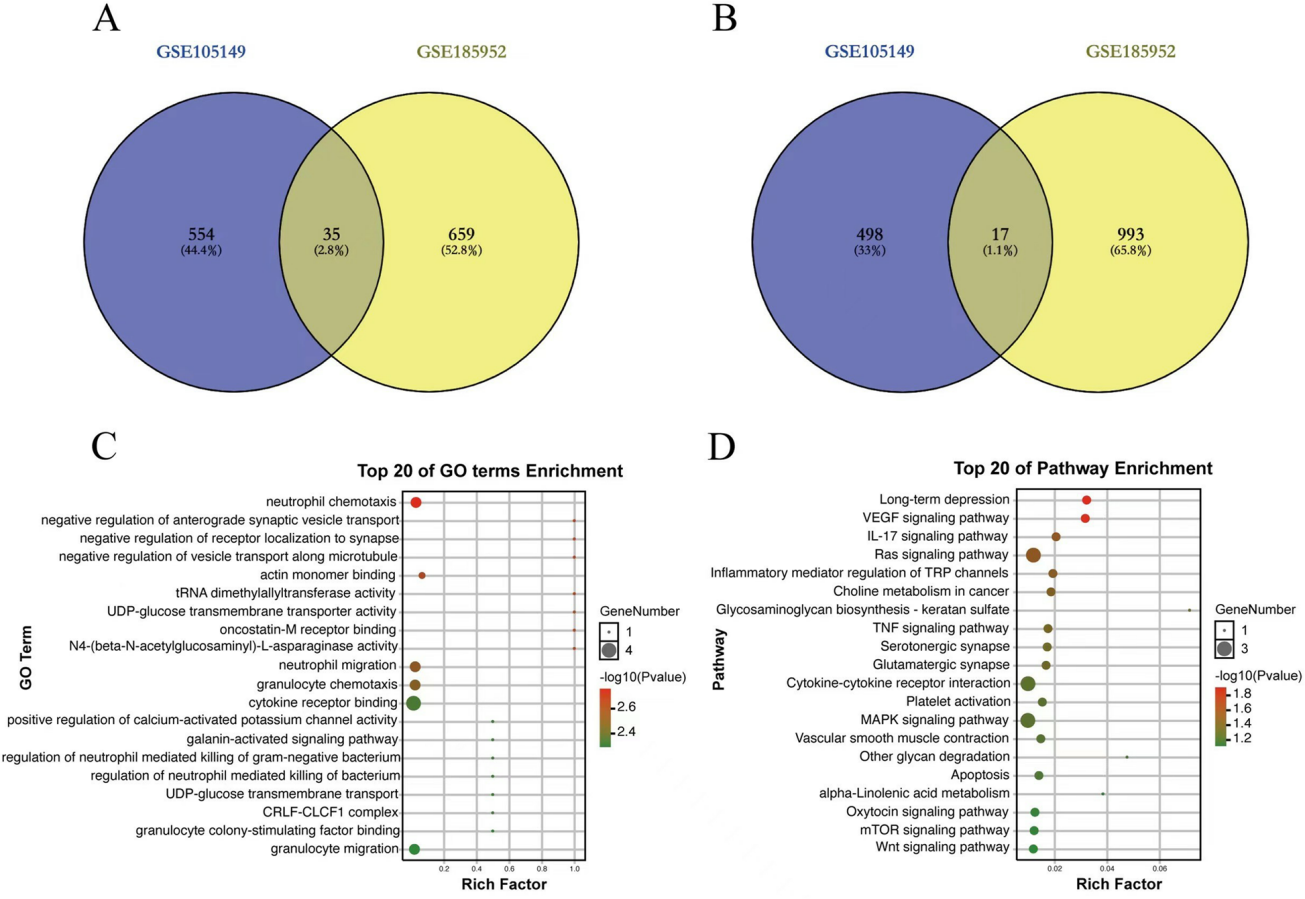
Identification and enrichment analysis of the common DEGs. **A** and **B** Venn diagrams showed the overlapping DEGs in the two datasets. **C** and **D** The bubble plots showed the top 20 GO and KEGG pathway enrichment in common DEGs

To further identify DEGs related to the lacrimal gland and orbital adipose/connective tissue in TAO patients, we screened common DEGs in both GSE105149 and GSE185952. All 52 DEGs, including 35 up-regulated and 17 down-regulated genes, were screened, as shown in Veen diagrams (Fig. 4A, B). The full names and functions of these 52 DEGs are provided in Table 1. GO enrichment and KEGG pathway analysis were performed based on the 52 DEGs, and the results indicated several immune response, neuromodulation, and cytokine-related biological processes to be enriched; the results shown in the bubble plot in Fig. 4C, such as neutrophil chemotaxis, negative regulation of vesicle transport along microtubules and cytokine receptor binding. Similarly, KEGG pathway analysis indicated striking enrichment in the immune, neuromodulation, and cytokine-related pathways (Fig. 4D), including long-term depression, the vascular endothelial growth factor (VEGF) signaling pathway, the IL-17 signaling pathway, the TNF signaling pathway and cytokine-cytokine receptor interactions, among the DEGs.

**Table 1:** The results of KEGG enrichment analysis of common DEGs

Pathway ID	KEGG Class	Pathway	*P* value	Genes
ko04730	Nervous system	Long-term depression	0.01252	PLA2G4D; PRKCG
ko04370	Signal transduction	VEGF signaling pathway	0.01291	PLA2G4D; PRKCG
ko04657	Immune system	IL-17 signaling pathway	0.02908	CXCL6; IKBKB
ko04014	Signal transduction	Ras signaling pathway	0.03053	PLA2G4D; IKBKB; PRKCG
ko04750	Sensory system	Inflammatory mediator regulation of TRP channels	0.03249	PLA2G4D; PRKCG
ko05231	Cancers	Choline metabolism in cancer	0.03484	PLA2G4D; PRKCG
ko00533	Glycan biosynthesis and metabolism	Glycosaminoglycan biosynthesis—keratan sulfate	0.03808	CHST6
ko04668	Signal transduction	TNF signaling pathway	0.03911	CXCL6; IKBKB
ko04726	Nervous system	Serotonergic synapse	0.04037	PLA2G4D; PRKCG
ko04724	Nervous system	Glutamatergic synapse	0.04228	PLA2G4D; PRKCG
ko04060	Signaling molecules and interaction	Cytokine-cytokine receptor interaction	0.04915	OSM; CXCL6; CSF3R
ko04611	Immune system	Platelet activation	0.04958	PLA2G4D; GP9
ko04010	Signal transduction	MAPK signaling pathway	0.04995	PLA2G4D; IKBKB; PRKCG

### Construction of the PPI network and identification of hub genes

**Fig. 5:**
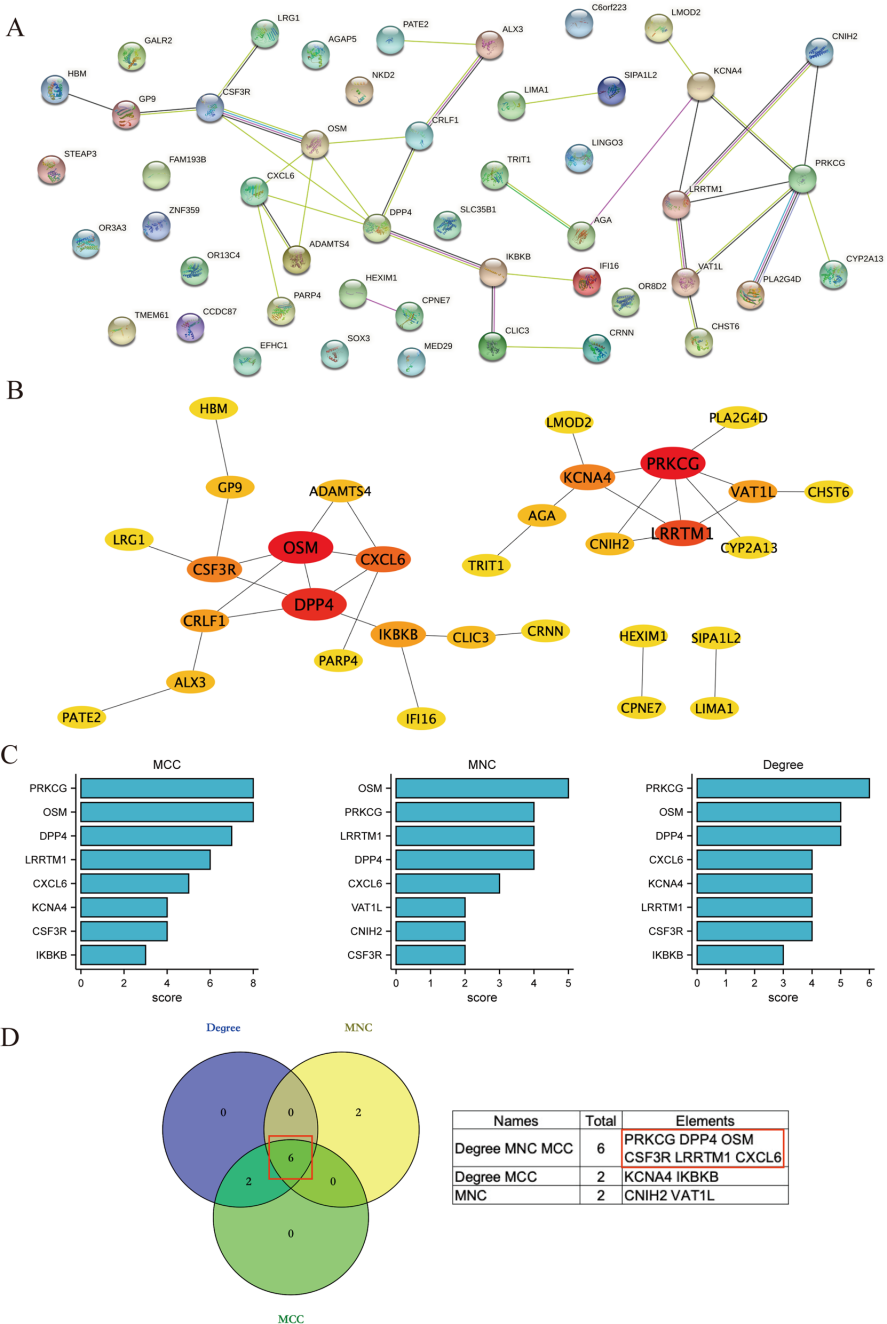
Construction of PPI network and identification of hub genes. **A** PPI network constructed by STRING to indicate the interactions among these 52 common DEGs. **B** PPI network of 52 common DEGs was constructed by Cytoscape. The different colors in the figure indicate the connectivity of genes, and the deep color shows the genes with the highest connectivity in the PPI network. **C** Screening the top 8 genes in the PPI network of DEGs as central genes using three algorithms including MCC, MNC, and Degree. **D** Venn diagram showed six common genes of three algorithms

To unravel protein interactions among the 52 DEGs and screen the hub genes in TAO, we submitted these common DEGs to the STRING database and constructed a PPI network using Cytoscape software (Fig. 5A, B). Then, three key algorithms (MCC, MNC, and ﻿Degree) of the CytoHubba application were used to screen the top 8 genes shown in bar plots (Fig. 5C). Afterwards, we intersected the results of the three algorithms and identified six hub genes (Fig. 5D): PRKCG, OSM, DPP4, leucine rich repeat transmembrane neuronal 1 (LRRTM1), CXCL6, and CSF3R.

### Validation of hub genes in clinical samples

**Fig. 6:**
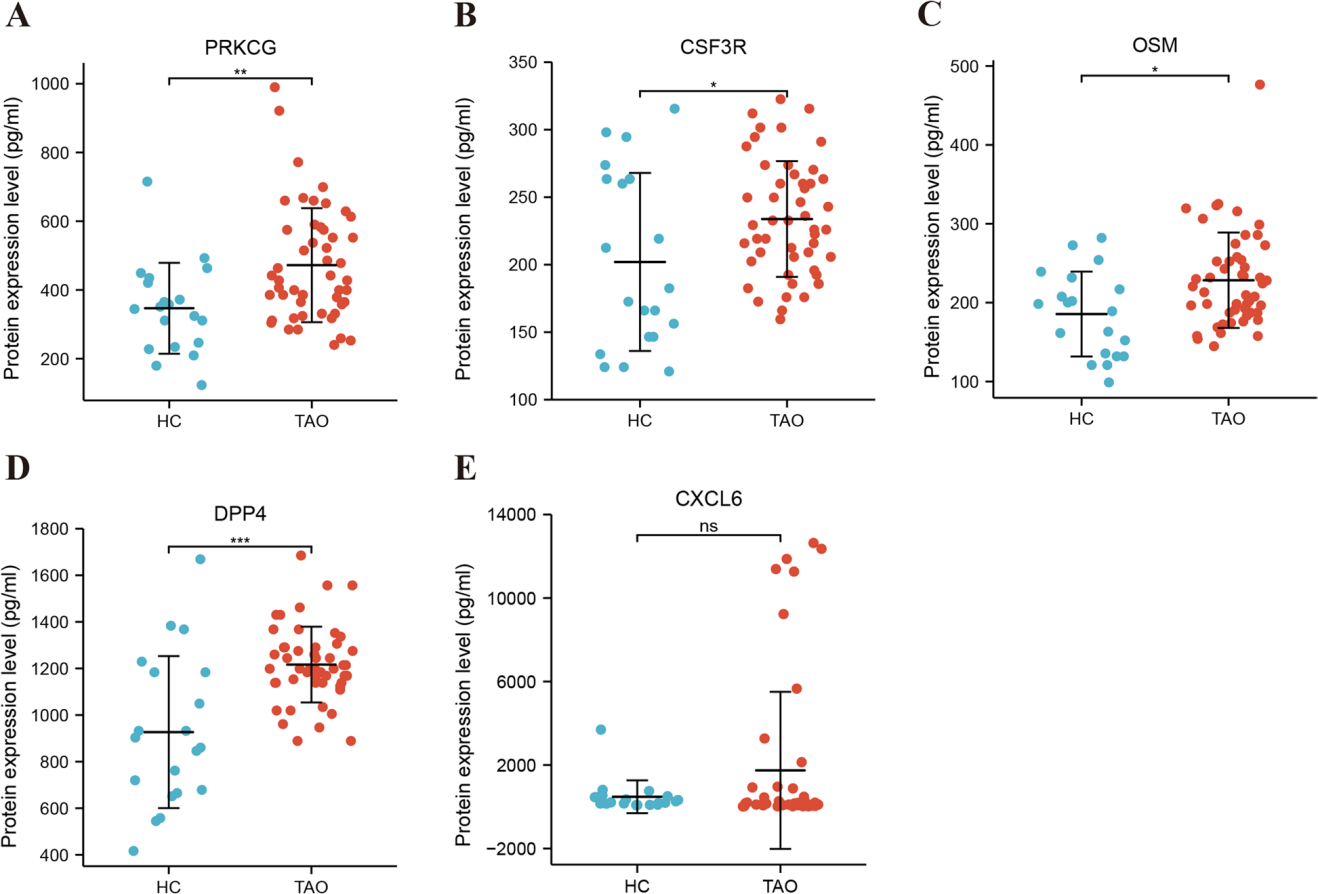
Validation of the relative protein expression levels by ELISA. Serum PRKCG, OSM, DPP4, CXCL6, and CSF3R expression levels in 50 TAO patients and 20 HCs was detected by ELISA. *P < 0.05, **P < 0.01, ***P < 0.001, ns, no significance

**Fig. 7:**
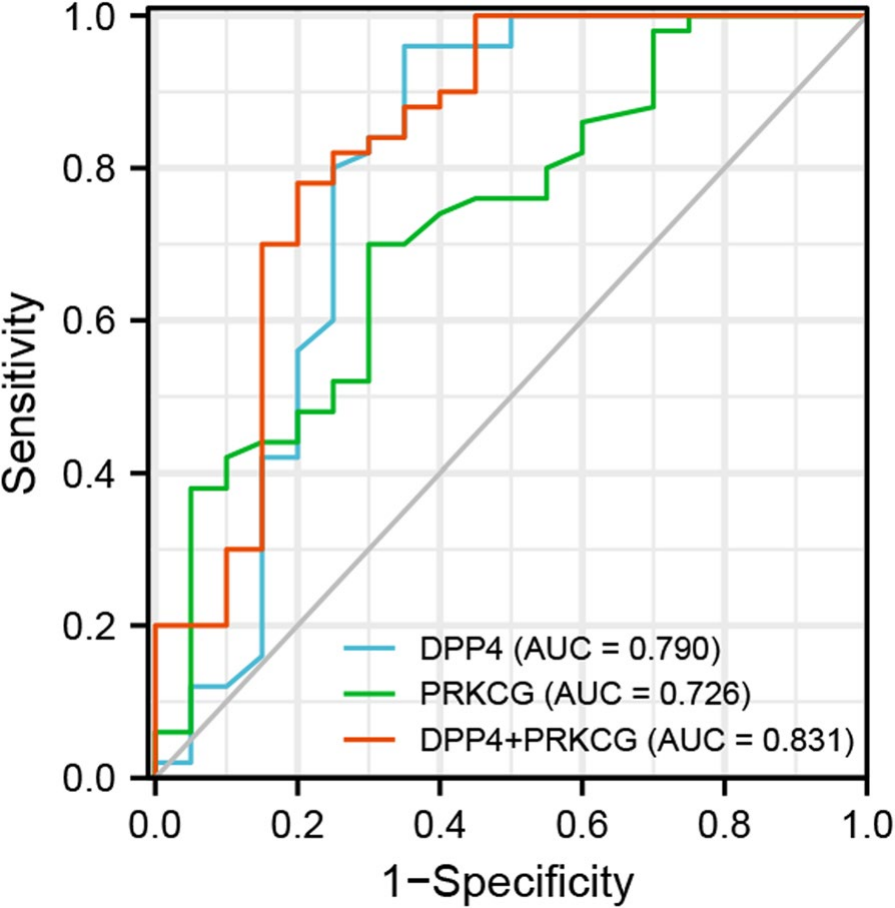
ROC curves show the diagnostic performance of DPP4 and PRKCG in identifying TAO patients

To verify the protein expression levels of PRKCG, OSM, DPP4, LRRTM1, CXCL6, and CSF3R, 70 serum samples from 20 healthy controls (HCs) and 50 TAO patients (patient characteristics shown in Table 2) were collected to perform ELISA. Since no commercial ELISA kit is available to detect serum LRRTM1, this target was not validated. The results indicated that compared with HCs, protein expression levels of PRKCG, OSM, CSF3R, and DPP4 were significantly up-regulated in TAO patients (*P* = 0.003, 0.016, 0.039 and 0.001, respectively, Fig. 6). CXCL6 exhibited a tendency towards higher levels in TAO patients, though without reaching statistical significance. To further evaluate the effectiveness and applicability of these genes in TAO, we performed multivariate and ROC analyses. Multivariate logistic regression analysis revealed DPP4 (*P* = 0.002; OR, 1.007; 95% confidence interval [CI], 1.003–1.011) and PRKCG (*P* = 0.017; OR, 1.008; 95% CI, 1.001–1.014) to be significantly associated with TAO occurrence. ROC analyses showed that PRKCG (AUC: 0.726, 95% CI: 0.592–0.861) and DPP4 (AUC: 0.790, 95% CI: 0.639–0.942) have good diagnostic value for TAO. The detailed ROC curve analysis results are depicted in Fig. 7. The combined AUC of PRKCG and DPP4 reached 0.831 (95% CI: 0.706–0.956), with the best specificity.

**Table 2:** Baseline characteristics of the study groups

Variables	HCs	All TAO	Active TAO	Inactive TAO
Number of individuals	20	50	39	11
Age (years)	41.60 ± 12.95	45.40 ± 12.68	46.85 ± 12.76	40.27 ± 11.46
Sex (F/M)	14/6	33/17	23/16	10/1
Smoking, n (%)	-	38	46.2	9.1
Disease duration (months)	-	5.92 ± 5.10	6.10 ± 4.55	5.27 ± 6.96
CAS	-	3.36 ± 1.14	3.79 ± 0.86	1.82 ± 0.41
FT3 (pmol/L)	-	6.16 ± 3.82	6.47 ± 4.25	5.07 ± 1.11
FT4 (pmol/L)	-	18.32 ± 9.78	18.86 ± 10.95	16.41 ± 2.72
TSH (mIU/L)	-	1.87 ± 2.49	1.88 ± 2.60	1.81 ± 2.14
TRAb (IU/L)	-	8.99 ± 11.34	9.37 ± 11.21	7.63 ± 12.24

The numeric data are reported as the mean ± standard deviation

*TAO* thyroid-associated ophthalmopathy, *F* female, *M* male, *CAS* clinical activity score, *FT4* free thyroxine, *FT3* free triiodothyronine, *TSH* thyroid-stimulating hormone, *TRAb* thyroid-stimulating hormone receptor antibody

### Association between the hub genes and TAO clinical information

**Fig. 8:**
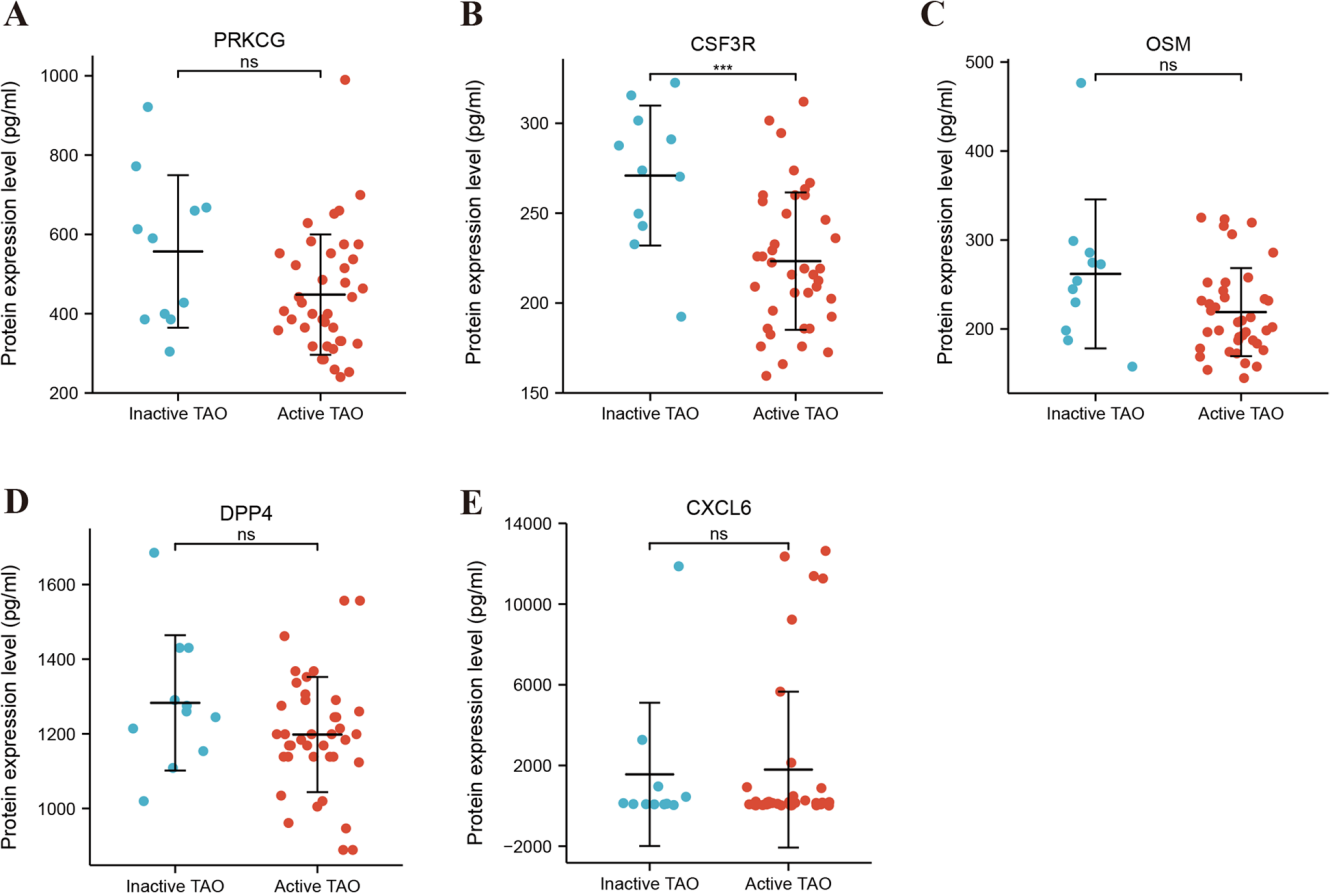
Comparisons of serum PRKCG, OSM, DPP4, CXCL6 and CSF3R expression levels between the active and inactive TAO patients. ***P < 0.001, ns, no significance

**Fig. 9:**
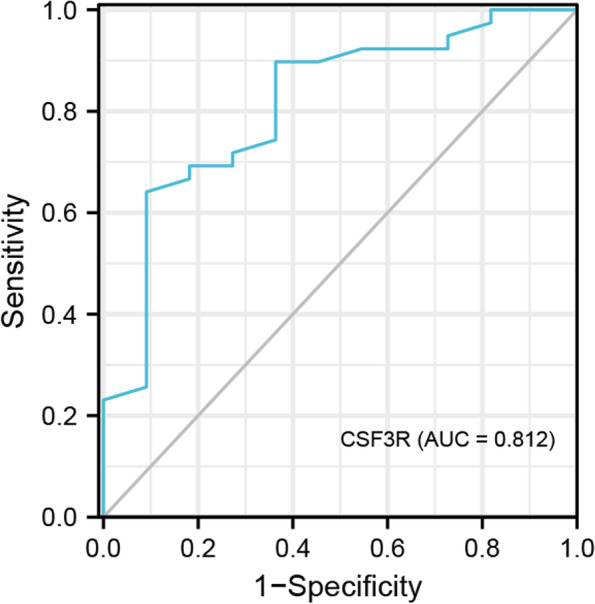
ROC curves of CSF3R in differentiating active TAO from inactive TAO patients

The association between the protein levels of CSF3R, CXCL6, DPP4, OSM, and PRKCG and clinical characteristics of the patients were analyzed by the Spearman correlation test (Supplementary Table 1). Our results showed that CSF3R protein levels presented a negative relationship with disease duration (*r* = -0.316, *P* = 0.025) and CAS (*r* = -0.45, *P* = 0.001). The DPP4 protein level was associated with high-density lipoprotein cholesterol (HDL-C) (*r* = -0.305, *P* = 0.031), CSF3R (*r* = 0.499, *P* < 0.001), and OSM genes (*r* = 0.323, *P* = 0.022). In addition, OSM protein expression was positively associated with the CSF3R gene (*r* = 0.526, *P* < 0.001). These correlations suggest that the hub genes may strongly interact with each other, which was consistent with the protein–protein interaction network. Furthermore, CSF3R was significantly up-regulated in active TAO patients when combined with clinical information (*P* = 0.001, Fig. 8), and the ROC curve (Fig. 9) showed that CSF3R has high diagnostic value in distinguishing active from inactive TAO patients (AUC = 0.812, 95%CI: 0.663–0.962).

## Discussion

TAO is a complex autoimmune disease, and its precise molecular mechanisms have not been completely elucidated; moreover, effective treatment options for TAO are limited [24]. Therefore, it is worth exploring novel biomarkers and therapeutic targets to improve the prognosis of TAO patients. With the application of bioinformatics, some studies have been performed to elucidate the pathogenesis of TAO. However, overreliance on public datasets and sequencing technology may destabilize results. In addition, most studies to date have focused on transcriptional regulation in a single tissue and the similarity and difference in transcription regulation among different lesion tissues are relatively unknown. In the present study, considering the tissue-specific effects, two public gene expression profiles containing different lesion tissues (lacrimal gland and orbital adipose/connective tissue) from TAO patients (GSE105149 and GSE185952) were used to screen and investigate key genes and pathways to examine the biological and clinical implications of TAO. Additionally, collected the serum samples of 50 TAO patients and 20 healthy controls to verify our findings through the ELISA. The obtained genes and pathways may provide novel insight into the underlying mechanisms and may reveal a new strategy for the diagnosis and treatment of TAO.

In this study, we separately analyzed the differentially expressed genes and pathways in the GSE105149 and GSE185952 datasets. To investigate the potential biological association between different lesion tissues in TAO, the results of functional enrichment analysis were compared, and three overlapping pathways (neuroactive ligand-receptor interaction, IL-17 signaling pathway, and TNF signaling pathway) were found between the two datasets. Therefore, it is logical to speculate that these three overlapping pathways may play key roles in TAO progression and development; other differential pathways might be influenced by tissue or organ specificity. In fact, consistent with our findings, several studies have confirmed that certain biomarkers and pathways, including cytokines, pro-inflammatory markers, and inflammation-related pathways, appear to be less specific to TAO [25]. Additionally, we sought to explain why the three overlapping pathways are not influenced by tissue specificity. Autoimmune responses are considered to be central in the development and progression of TAO. The IL-17 and TNF signaling pathways are inflammatory pathways associated with TAO [26–28]. Furthermore, preliminary studies have found that blocking IL or TNF-α pathways may constitute a systemic treatment strategy for TAO [29, 30]. This evidence suggests that the two inflammation-related pathways might be involved in the occurrence and development of TAO, which may account for the lack of influence of tissue specificity. Similarly, growing evidence shows that some neuromodulation-related pathways may be involved in TAO [31, 32]. Thus, inflammatory mechanisms and neuronal function may play important roles in the different lesion tissues of TAO. However, the exact mechanisms underlying these pathways remain unclear.

In the present study, a set of 52 common DEGs from the GSE105149 and GSE185952 datasets were identified, including 35 up-regulated genes and 17 down-regulated genes. To further explore the biological function of these 52 common DEGs, functional enrichment analysis was performed; long-term depression, the VEGF signaling pathway, the IL-17 signaling pathway, the Ras signaling pathway and inflammatory mediator regulation of transient receptor potential (TRP) channels were the top 5 pathways. The IL-17 and TNF signaling pathways showed enrichment, while the neuroactive ligand-receptor interaction pathway was not directly included. Other neuromodulatory signaling pathways were also enriched, such as serotonergic synapse and glutamatergic synapse pathways, which was indirectly verified by our findings. Surprisingly, long-term depression containing PLA2G4D and PRKCG genes was the top pathway, suggesting that this pathway and these genes may have novel potential biological implications in TAO. PLA2G4D, a member of the phospholipase A2 (PLA2) family, promotes production of lipid mediators and regulates normal physiology and disease pathogenesis in many organ systems [33]. One previous study confirmed that another three family members, PLA2G4A, PLA2G4B, and PLA2G4C, are strongly associated with mental illness in the Chinese population [34]. These findings add support to the results of our previous study using diffusion tensor imaging that TAO patients develop disruption of the structural brain network connectome, which may be associated with clinical-psychiatric dysfunction in TAO [35]. Previous studies have also demonstrated that TAO patients are more prone towards emotional disorders, such as anxiety and depression [36, 37]. Nevertheless, the specific relationship between TAO and depression remains to be investigated.

In the present study, six key genes (PRKCG, OSM, CSF3R, LRRTM1, CXCL6, and DPP4) among 52 common DEGs were analyzed in the PPI network based on interactions. PRKCG encodes protein kinase C gamma (PKC gamma), a member of the serine/threonine kinase family, which is abundantly expressed in neural tissue [38]. Studies have shown that PKC dysregulation is associated with major depression [39, 40]. LRRTM1, an imprinted gene that affects neuronal differentiation and connectivity, correlates with schizophrenia/schizoaffective disorder [41]. However, the precise roles of PRKCG and LRRTM1 in TAO are unknown, and we suspected they may be involved in the mechanisms of the impaired emotional regulation and higher tendency to depression or anxiety occurring in TAO patients [3, 35]. In addition, four other key genes, CSF3R, OSM, CXCL6, and DPP4, showed positive associations with inflammatory processes in TAO. For example, CSF3R is the receptor for colony stimulating factor 3, a cytokine that controls expansion and differentiation of neutrophils. Mutations in CSF3R have been linked to chronic neutrophilic leukemia [42]. To our knowledge, no study to date has analyzed the effects of CSF3R in TAO. OSM is a proinflammatory cytokine that belongs to the interleukin-6 (IL-6) family. It is produced mainly by activated macrophages, neutrophils and mast cells [43]. Chen et al. discovered that IL-6 is overexpressed in the orbital tissue of patients with TAO, promoting proliferation and differentiation of B cells, and activation of T helper 1 (Th1) cells, releasing a variety of cytokines [44]. However, a possible role for OSM in TAO has not been reported. CXCL6, a member of the CXC chemokine family, is highly expressed in patients with diabetic nephropathy (DN) [45]. In an in vitro study, overexpression of CXCL6 significantly up-regulated expression of proinflammatory and profibrotic cytokines [46]. Of note, Antonelli et al. reported that interferon-gamma (IFN-gamma) and TNF-alpha stimulate release of chemokines, particularly CXCL9, CXCL10 and CXCL11, and induce recruitment of activated T cells to maintain inflammation in TAO [47]. Whether CXCL6 has similar roles in TAO remains unknown. In fact, proinflammatory cytokines have been extensively studied in recent years in relation to the pathogenesis of TAO [48, 49], and our functional enrichment results indicated that OSM, CXCL6 and CSF3R genes are all closely related to the cytokine-cytokine receptor interaction pathway. It will be helpful to further clarify their specific roles in the inflammatory process. DPP4, also known as CD26, is a cell surface glycoprotein receptor that plays an important role in regulating T-cell activation. Previous studies have indicated that IFN-gamma and IL-12 can facilitate expression of CD26 on CD4 ^+^ T cells, thereby promoting Th1-like cytokine production [50]. It is well known that in the early stage of TAO production of pro-inflammatory cytokines by Th1 cells can enhance fibroblast proliferation and glycosaminoglycan production. Therefore, we speculate that DPP4 might be involved in the pathogenesis of TAO.

In the current study, PRKCG, OSM, CSF3R, LRRTM1, and CXCL6 were up-regulated and DPP4 was down-regulated in TAO. Considering the limitations of public datasets and bioinformatics, we verified the results using clinical samples from our center. As no commercial ELISA kit was available for testing serum LRRTM1, only PRKCG, OSM, CSF3R, CXCL6 and DPP4 protein levels were detected via ELISA in 50 TAO patients and 20 HCs. Overall, protein expression levels of PRKCG, OSM, and CSF3R were significantly higher in TAO patients, consistent with the bioinformatics results. Similarly, protein expression level of CXCL6 exhibited an up-regulated tendency in TAO, though the difference was not significant. This may be due to individual variation in CXCL6 expression among TAO patients. Interestingly, the mRNA and protein expression levels of DPP4 seem to be contradictory, as DPP4 protein expression was upregulated in TAO patients, but mRNA expression was decreased, which may be ascribed to the non-strict linear relationship between mRNA and protein as well as more complex dependencies. For example, different regulatory mechanisms (RNA modifications, RNA-RNA interactions, synthesis and degradation rates) might explain the different results in DPP4 mRNA and protein expression levels [51], and more samples are needed to explore this possibility. According to multivariable regression, we further found that the expression levels of DPP4 and PRKCG were significantly associated with TAO occurrence. The area under the ROC curve illustrated that these two genes exhibit good diagnostic value for TAO. Additionally, CSF3R is a promising candidate for distinguishing between active TAO patients and inactive ones. Therefore, these hub genes may play important roles in the pathogenesis of inflammatory processes, with great potential as reliable and robust blood diagnostic biomarkers and therapeutic targets for TAO. In summary, the novelty of this study mainly includes the following aspects: First, to the best of our knowledge, the six genes (PRKCG, OSM, DPP4, LRRTM1, CXCL6, and CSF3R) identified are for the first time reported to be differentially expressed in various samples (lacrimal gland and orbital adipose/connective tissue) of TAO patients and defined as hub genes for TAO; Second, we first report the interactions of these hub genes in TAO patients, and these hub genes might be associated with disease progression and clinical characteristics of TAO; Third, bioinformatics analysis combined with molecular biology was used to explore potential genes and pathways in TAO patients, and the obtained genes and pathways provide novel insight into the underlying mechanisms and may reveal a new strategy for diagnosis and treatment of TAO.

However, there are still some limitations and prospects in our study. First, although the latest datasets for different lesion tissues of TAO were selected, the sample size was small, and the results need to be confirmed in larger samples; Second, as the current work was based on omics-research and focused on marker screening, few functional studies of these biomarkers were performed. Therefore, it is necessary to further explore the exact mechanisms of TAO influenced by these biomarkers; Third, multiple studies have confirmed the value of long noncoding RNAs (lncRNAs) and microRNAs (miRNAs) in disease development, prediction and treatment [52, 53], and several lncRNA- and miRNA-disease associations might be identified by novel state-of-the-art computational models [54–56]. However, our study did not cover these aspects. Elaborating on such connections will be a good direction for future research.

## Conclusion

In summary, the genes OSM, CSF3R, CXCL6, and DPP4-induced inflammatory processes may participate in the pathogenesis of TAO. Combined with ROC analyses, the results illustrate that the DPP4 and PRKCG gene exhibit diagnostic value for TAO and that CSF3R can effectively discriminate disease activity. Another intriguing finding is that PRKCG and LRRTM might be associated with mental disorders in TAO, providing a novel mechanistic explanation for clinical mental disorders occurring in TAO patients. Taken together, these findings partially unravel the mechanisms of TAO pathogenesis, which will be beneficial for TAO diagnosis and treatment.

## Supplementary Information

**Additional file 1: Supplementary Table 1. **Correlations between clinical characteristics and protein expression levels of the study subjects.

## Data Availability

The datasets generated and/or analyzed during the current study are available in the Gene Expression Omnibus database (http://www.ncbi.nlm.nih.gov/geo).

## Abbreviations

TAO: Thyroid-associated Ophthalmopathy
GEO: Gene Expression Omnibus
DEGs: Differential expressed genes
GO: Gene Ontology
KEGG: Kyoto Encyclopedia of Genes and Genomes
PPI: Protein-protein interaction
HCs: Healthy controls
ELISA: Enzyme-linked immunosorbent assay
FC: Fold change
BP: Biological processes
CC: Cellular component
MF: Molecular function
STRING: Search Tool for the Retrieval of Interacting Genes
MCC: Maximal Clique Centrality
MNC: Maximum Neighborhood Component
PRKCG: Protein Kinase C Gamma
OSM: Oncostatin M
DPP4: Dipeptidyl Peptidase 4
CXCL6: C-X-C Motif Chemokine Ligand 6
CSF3R: Colony Stimulating Factor 3 Receptor
LRRTM1: Leucine Rich Repeat Transmembrane Neuronal 1
TNF: Tumor necrosis factor
ROC: Receiver operating characteristic
AUC: Area under the curve
VEGF: Vascular endothelial growth factor
TRP: Transient receptor potential
PLA2: Phospholipase A2
PKC gamma: Protein kinase C gamma
IL-6: Interleukin-6
TH1: T helper 1
