# Supplementary material for: Comprehensive analysis of key genes and pathways for biological and clinical implications in thyroid-associated ophthalmopathy
Source: BMC Genomics. 2022 Sep 2;23:630. doi: 10.1186/s12864-022-08854-5 (PMC9440526; doi:10.1186/s12864-022-08854-5)
Supplement: Supplementary file 1 — Additional file 1: Supplementary Table 1. Correlations between clinical characteristics and protein expression levels of the study subjects. [file 12864_2022_8854_MOESM1_ESM.docx]

**Supplementary Table 1** Correlations between clinical characteristics and protein expression levels of the study subjects

|  | CSF3R | | CXCL6 | | DPP4 | | OSM | | PRKCG | |
| --- | --- | --- | --- | --- | --- | --- | --- | --- | --- | --- |
| parameters | coefficient | *P* | coefficient | *P* | coefficient | *P* | coefficient | *P* | coefficient | *P* |
| age (years) | 0.032 | 0.823 | 0.141 | 0.328 | -0.093 | 0.522 | 0.203 | 0.157 | 0.020 | 0.892 |
| gender | -0.110 | 0.448 | 0.064 | 0.657 | -0.070 | 0.627 | -0.022 | 0.880 | 0.176 | 0.222 |
| Disease duration (months) | -0.316 | 0.025 | 0.049 | 0.734 | -0.057 | 0.697 | -0.217 | 0.130 | -0.267 | 0.061 |
| CAS | -0.450 | 0.001 | -0.155 | 0.282 | -0.226 | 0.114 | -0.228 | 0.112 | -0.207 | 0.149 |
| FT3 (pmol/L) | 0.071 | 0.625 | -0.061 | 0.674 | 0.019 | 0.898 | -0.003 | 0.983 | -0.022 | 0.878 |
| FT4 (pmol/L) | -0.031 | 0.833 | 0.067 | 0.642 | -0.079 | 0.584 | 0.056 | 0.701 | -0.032 | 0.825 |
| TSH (mIU/L) | -0.220 | 0.125 | 0.093 | 0.52 | -0.034 | 0.815 | -0.188 | 0.190 | 0.041 | 0.777 |
| TRAb (IU/L) | 0.019 | 0.897 | -0.106 | 0.462 | 0.0630 | 0.664 | -0.059 | 0.683 | 0.079 | 0.585 |
| total cholesterol (mg/dL) | -0.122 | 0.401 | 0.156 | 0.279 | -0.261 | 0.067 | 0.015 | 0.917 | 0.021 | 0.883 |
| TG (mg/dL) | -0.051 | 0.723 | 0.036 | 0.806 | 0.021 | 0.883 | -0.255 | 0.074 | 0.029 | 0.839 |
| HDL-C (mg/dL) | -0.019 | 0.898 | -0.214 | 0.136 | -0.305 | 0.031 | 0.066 | 0.647 | -0.026 | 0.860 |
| LDL-C (mg/dL) | -0.027 | 0.854 | 0.254 | 0.076 | -0.017 | 0.906 | 0.207 | 0.148 | 0.086 | 0.552 |
| CSF3R | NA | NA | -0.265 | 0.063 | 0.499 | <0.001 | 0.526 | <0.001 | 0.093 | 0.521 |
| CXCL6 | -0.265 | 0.063 | NA | NA | 0.050 | 0.732 | 0.102 | 0.482 | -0.042 | 0.770 |
| DPP4 | 0.499 | <0.001 | 0.050 | 0.732 | NA | NA | 0.323 | 0.022 | 0.076 | 0.601 |
| OSM | 0.526 | <0.001 | 0.102 | 0.482 | 0.323 | 0.022 | NA | NA | 0.043 | 0.768 |
| PRKCG | 0.093 | 0.521 | -0.042 | 0.770 | 0.076 | 0.601 | 0.043 | 0.768 | NA | NA |

CAS, clinical activity score; FT3, free triiodothyronine; FT4, free thyroxine; TSH, thyroid-stimulating hormone; TRAb, thyroid-stimulating hormone receptor antibody; TG, triglyceride; HDL-C, high-density lipoprotein cholesterol; LDL-C, low-density lipoprotein cholesterol.

Statistical significance is indicated by *p* values < 0.05
